# Cremation in Its Legal Aspects

**Published:** 1885-07

**Authors:** 


### Cremation in its Legal Aspects.

Cremation is on the increase in this country and in England, and renewed attention is being called to the matter by some recent notable instances where this mode of disposing of the bodies of the dead was adopted in preference to burial. The fact that there is but one crematory in the country, that at Washington, Pa., and the impossibility of sending corpses there conveniently and economically, from other parts of the country, will prevent any extensive use of cremation, even by those who favor it.

The vast majority of the community, however, are still emphatically in favor of burial, and will, no doubt, resist any change on sentimental and religious grounds.

The legal status of the question is somewhat unsettled, but it is doubtful if any action taken in our American courts could prevent any persons from cremating a dead body, who wished to do so, and it was not contrary to the expressed wishes of the deceased. The law books speak of "Christian burial" as a right of every person, but this was declared at a time when cremation was not thought of, and the expression would probably not avail in opposition to an attempt to cremate a body. There have been two cases in England recently, which will be interesting. In one decided about two years ago, a person named H. Crookenden directed his friend, Eliza Williams, to burn his body, and directed his executors to pay the expenses. The executors, however, buried the body, and Miss Williams obtained permission to disinter it, in order to bury it elsewhere as she said. When it was once in her possession, she proceeded to cremate it, and then sued the executors for her expenses.

In this suit she was beaten, the court holding that the removal of the body, and the burning of it under the circumstances was illegal, having been accomplished by misrepresentations. The judge stated expressly that he would not decide whether burning the body was in itself legal or illegal under the laws of England.

That question has just been presented, however, in a case tried at Cardiff by Justice Stephen, and decided, after a careful examination of the whole matter, in favor of the legality of cremation. The circumstances of the case were quite peculiar. A man named Price had a child five months old, which died without the fact of disease being registered as required by law. The coroner then gave him notice that unless the proper certificate was filed by a certain day, an inquest would be held. On the day when this inquest was to be held Price took the body of the child to a field belonging to him, placed it in a cask of petroleum and set the oil on fire. A crowd quickly collected, extinguished the fire by covering it with earth, and Price was arrested. An indictment was subsequently found on the two charges committing a misdemeanor by cremating the body, and by preventing an inquest. The judge, in determining the case, said that after a full examination of the law it could not be held that cremation in itself was a misdemeanor, as Parliament had never declared it to be such, and that judges without such previous action, would not declare any act to be a misdemeanor which was not at the same time a public nuisance by reason of its annoying or injuring others. The grand jury were directed to inquire into the facts, and see if the act of Price was so public and annoying as to make it a misdemeanor. The grand jury, as has been stated, did find an indictment for this reason, but on the subsequent trial the defendant was acquitted. He was also acquitted on the other ground of preventing an inquest, on the ground, probably, although the report does not fully inform the public, that the coroner did not have sufficient grounds for holding an inquest, and consequently any action preventing an inquest was not illegal. It must be remembered that coroners have not the right to hold inquests at their own will, and they become liable to the law if they interfere with funerals where there are no suspicious circumstances attending the death. The jury, it would seem did not believe that there were any suspicious circumstances connected with the death of the child. and therefore Price did not do wrong in burning the body, this act not being in itself illegal.

The court court discussed also the question whether every corpse was entitled to "Christian burial" and said that the acts allowing the use of certain bodies for anatomical purposes, showed that it was not a universal right, aside from the fact that the expression was used without thought of cremation.—*Therapeutic Gazette.*

---

Parke, Davis & Co. have furnished us a convenient and tastefully arranged case enclosing Oliver's urinary test papers, with instructions for their use. They are designed for the clinical examination of urine at the bed-side. The case contains litmus paper, which serves as a test for acidity or alkalinity of the secretion; as tests for albumen, the case includes four of the recently introduced reagents: 1, picric acid; 2, potassio-mercuric iodide; 3, potassium-ferro-cyanide; 4, sodium tungstate, all of which are to be used in connection with citric acid. As tests for sugar, the series of test papers includes, first, a modification of the usual copper test, and, second, picric acid in combination with carbonate of sodium. For use by the physician, especially in the country, where chemicals are not easy of access, this compact arrangement meets a want which has been long felt. We have used them with success and satisfaction. The case contains, also, a graduated pipette and specific gravity beads, with full directions for their use. We direct attention to this case with a view to commend them to many in the profession who have neither the time nor the taste to convert their office into a chemical laboratory for analytical investigations, and who need just such a compact test case to take with them in their daily practice.

